# Seroprevalence Survey of Anti-SARS-CoV-2 Antibodies in a Population of Emilia-Romagna Region, Northern Italy

**DOI:** 10.3390/ijerph19137882

**Published:** 2022-06-27

**Authors:** Stefania Paduano, Pasquale Galante, Nausicaa Berselli, Luca Ugolotti, Alberto Modenese, Alessandro Poggi, Marcella Malavolti, Sara Turchi, Isabella Marchesi, Roberto Vivoli, Paola Perlini, Rossana Bellucci, Fabriziomaria Gobba, Marco Vinceti, Tommaso Filippini, Annalisa Bargellini

**Affiliations:** 1 Department of Biomedical, Metabolic and Neural Sciences, University of Modena and Reggio Emilia, 41125 Modena, Italy; pasquale.galante1991@libero.it (P.G.); nausicaa.berselli@unimore.it (N.B.); 212164@studenti.unimore.it (L.U.); alberto.modenese@unimore.it (A.M.); alle.poggi.90@gmail.com (A.P.); marcella.malavolti@unimore.it (M.M.); sara.turchi@unimore.it (S.T.); isabella.marchesi@unimore.it (I.M.); fabriziomaria.gobba@unimore.it (F.G.); marco.vinceti@unimore.it (M.V.); tommaso.filippini@unimore.it (T.F.); annalisa.bargellini@unimore.it (A.B.); 2 Test Laboratory, 41100 Modena, Italy; vivoli.roberto@laboratoriotest.it (R.V.); mdl@laboratoriotest.it (P.P.); bellucci.rossana@laboratoriotest.it (R.B.); 3 Department of Epidemiology, Boston University School of Public Health, Boston, MA 02118, USA; 4 School of Public Health, University of California Berkeley, Berkeley, CA 94704, USA

**Keywords:** COVID-19, occupational risk, SARS-CoV-2, seroprevalence, waves, workers

## Abstract

Italy was the first Western European country to be severely hit by the COVID-19 pandemic. Variations in seroprevalence rates were reported according to geographical and temporal differences of previous surveys, as well as depending on demographic and occupational factors. In this cross-sectional study, we evaluated the prevalence of anti-SARS-CoV-2 antibodies in a population of the Emilia-Romagna region in Northern Italy after the first wave in the period from 26 September 2020–26 March 2021. We included 5128 subjects who voluntarily underwent serological tests to determine anti-SARS-CoV-2 antibody positivity, including both self-referred individuals (24.2%) and workers adhering to company screening programs (76.8%). Overall, seroprevalence was 11.3%, higher in self-referred (13.8%) than employed-referred (10.5%) individuals. A slightly higher seroprevalence emerged in women compared to men (12.3% and 10.7%), as well as in the extreme age categories (18.6% for 60–69 years, 18.0% for ≥70 years, and 17.1% for <20 years compared to 7.6% for 20–39 years). Healthcare professionals showed the highest prevalence of seropositivity (22.9%), followed by workers in direct contact with customers, such as the communication, finance, and tourism sectors (15.7%). Overall subgroups seroprevalence increased compared to the first wave data but the trends agreed between the first and subsequent waves, except for an increase in the younger age group and in the sector in direct contact with customers. Among the occupational categories, our study confirms that healthcare workers and workers in the sports sector were at high risk of exposure to SARS-CoV-2.

## 1. Introduction

Italy was the first Western European country severely affected by SARS-CoV-2 infection, with the first case diagnosed in February 2020, followed by a rapid spread of the virus, especially in North of the country [1].

Considering the public health emergency and the international concern, the WHO declared the outbreak a pandemic on 11 March 2020 and, after two years, more than 515 million infections and 6.2 million of deaths occurred worldwide [2]. In Italy, more than 16.6 million infections and over 164 thousand deaths were reported in two years [3]. The distribution was uneven across the country; especially in the first wave, the Northern regions were most affected [4]. The tight mobility restrictions (the lockdown) and the testing and tracing measures were essential for the decline of the spread of SARS-CoV-2 after the first wave, in June and July 2020 [5,6], due to the limited effective and specific therapies [7,8] and before the availability of an effective vaccination [9,10].

SARS-CoV-2 infection can be identified through diagnostic molecular RT-PCR test which are collected via nasopharyngeal or oropharyngeal swab [11,12]. Nonetheless, molecular testing campaigns cannot reflect the overall number of infected individuals, especially in the first wave of the epidemic, when the number of performed tests was low [13]. Therefore, seroprevalence of anti-SARS-CoV-2 antibodies may allow the identification of the undetected asymptomatic or pauci-symptomatic individuals, in particular, as well as the population groups with higher risk of infection [14,15]. Considering that, some previous studies used seroprevalence data in order to better understand the distribution and the severity of SARS-CoV-2 infection [13,16]. The national average seroprevalence after the first wave was estimated to be approximately 2.5%, with the highest values and variation in Northern Italy, depending on the province of residence [13,17]. In particular, a seroprevalence study carried out in a large, highly affected area located in Northeastern Italy after the first wave reported an IgG seroprevalence of 23.1% (95% confidence interval-CI 22.0–24.1%) [14]. Some studies investigated seroprevalence among workers, noting that the most affected were healthcare workers (5.6%), confirming the occupational risk for both symptomatic and asymptomatic infections [18], followed by those involved in the food sector (4.2%) [19,20] and workers in close contact with the general public [21,22].

Considering these factors, the estimation of seroprevalence rates within a specific population remains challenging but still relevant for the evaluation of the number of previous SARS-CoV-2 infections and the current immunity level, which are fundamental for the understanding the risk of disease transmission and the effectiveness of the strategies required to prevent it [14,23].

Following a previous study assessing the seroprevalence of anti-SARS-CoV-2 antibodies in subjects living in the central-western part of the Emilia-Romagna region before the second wave (1 June–25 September 2020) [17], in this survey, we aimed to evaluate the seroprevalence in the same area for the subsequent period with assessment and comparison of the characteristics of the study populations in order to identify potential risk factors that favored the infection development.

## 2. Materials and Methods

### 2.1. Study Population

We performed a cross-sectional study investigating the prevalence of anti-SARS-CoV-2 antibodies in a population living in the central-western part of the Emilia-Romagna region in the period between 26 September 2020 and 26 March 2021. This investigation follows a previous study assessing the seroprevalence of anti-SARS-CoV-2 antibodies in subjects of the same area in the period from 1 June–25 September 2020 [17]. To do so, we collected data from the Test laboratory located in Modena province, which is one of the first accredited laboratories for serological SARS-CoV-2 testing in the Emilia-Romagna region out of the only eight locations allowed to perform such tests during the study period (Decree PG/2020/0307727 of 22 April 2020). This laboratory was the only one in the province of Modena within approximately 80 km distance from other accredited laboratories at the beginning of the pandemic. After obtaining the Ethics Committee approval for the present study, we included all the adults tested for SARS-CoV-2 serological screening that were referred to the Test laboratory during the period between 26 September 2020 and 26 March 2021: workers referred by their companies, which recommended that their employees were tested, as well as self-referred individuals voluntarily admitted to the facility to undergo SARS-CoV-2 testing. No other selection criteria were considered. All subjects signed informed consent for sample collection and analysis.

### 2.2. Laboratory Analysis

Quantitative or qualitative tests were carried out to detect SARS-CoV-2 IgM/IgG antibodies in the serum of subjects, according to the participants’ preference. After receiving written consent, 5 mL of venous blood samples were drawn for quantitative tests or a drop of peripheral blood for qualitative tests. Concerning the quantitative analysis, the Elecsys^®^ Anti-SARS-CoV-2 test kit for IgG and IgM (Roche Diagnostics, Risch-Rotkreuz, Switzerland) was used, with 100% sensitivity 14 days after symptom onset and 99.8% specificity. For the qualitative analysis, the KHB^®^ diagnostic kit for SARS-CoV-2 IgM/IgG antibody Colloidal Gold was used, with 98.8% sensitivity and 98.0% specificity.

### 2.3. Data Analysis

Participants were defined as anti-SARS-CoV-2 antibody-positive (Ab+) when they were positive for IgM and/or IgG antibody.

In this paper, data are presented as mean and standard deviation for continuous variables and are shown in number and percentage (%) for categorical variables. Whenever possible, we performed subgroup analyses including sex, age (10-year categories), test type (quantitative vs. qualitative), Ig type (IgG vs. IgM), referral category (workers vs. private), and occupational status. For the latter, we used the 2007 ATECO classification [24] according the highest aggregation level considering the main 12 categories. Some activities involving mostly sedentary and office work (ATECO sections J, K, M, N) were merged into a single category. We used Microsoft Excel v.16 (2021—Microsoft Corporation, Reymond, WA, USA) and Stata software v. 16.1 (2021—Stata Corp., College Station, TX, USA) for data collection and analysis.

## 3. Results

Table 1 reports characteristics of the study participants. In the period between 26 September 2020 and 26 March 2021, 5128 individuals were tested for the presence of antibodies against SARS-CoV-2 with an overall age (mean ± standard deviation) of 43.5 ± 14.8 years, with 91.7% aged under 65. A total of 3124 (60.9%) were men and 2004 (39.1%) were women. Most of the participants resided in Modena (78.7% of all individuals, 76.6% of the men and 82.1% of the women), 114 (2.2%) resided in Reggio Emilia, 9 (0.2%) in Parma, 20 (0.4%) in Bologna, and the remaining 947 (18.5%) in other provinces. In the stratified analyses by age and province of residence, the distribution was comparable among the sexes. In the study, 3889 participants (76.8%) were workers undergoing testing for surveillance screening in the workplace, while 1239 (24.2%) came to the laboratory as private subjects. For the latter, no information on working conditions was therefore available. The most represented occupational sectors were “information and communication services/financial and insurance activities; etc.” (32.3%), “manufacturing activities” (30.4%), “health sector” (14.7%) and “sports activities” (11.3%).

The number of participants with a positive test for serum anti-SARS-CoV-2 antibodies was 580 (11.3%). The seroprevalence according to the province of residence was 9.4% for Modena (381 positive subjects), 25% for Bologna (5 positive subjects), 11.1% for Parma (1 positive subject), and 18.4% for Reggio Emilia (21 positive subjects).

**Figure 1:**
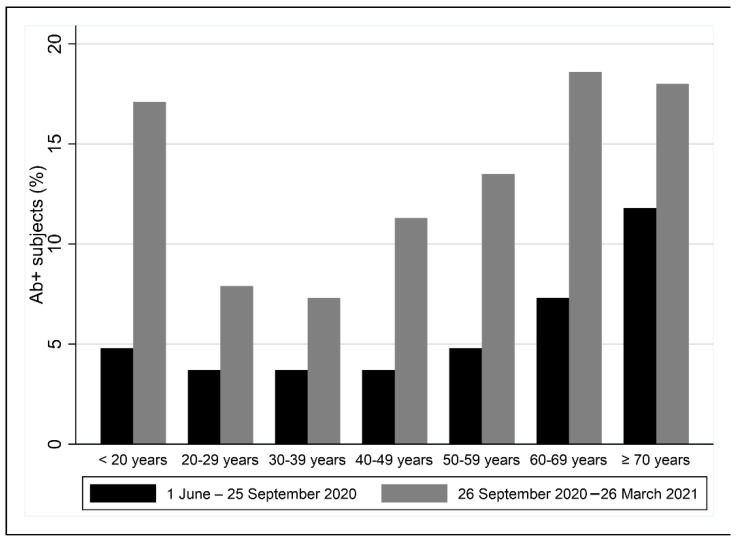
Percentage of positive subjects for anti-SARS-CoV-2 antibodies (Ab+) according to age class between the period 1 June–25 September 2020 and the subsequent period 26 September 2020–26 March 2021.

The seroprevalence is slightly higher in women than in men (12.3% vs. 10.7%), as shown in Table 2 and Table S1. The participants’ ages were higher in the seropositive subjects (47.6 ± 16.1 years) that in the negative ones (43.0 ± 14.6 years). In fact, the two oldest groups showed the highest seroprevalence: 18.0% and 18.6% in participants aged ≥70 and 60–69 years, respectively. Moreover, seroprevalence is also high among younger subjects aged <20 years (17.1%). Figure 1 shows a comparison of positive subjects (%) for anti-SARS-CoV-2 antibodies according to age class in the period 1 June–25 September 2020 [17] and the subsequent period 26 September 2020–26 March 2021.

A total of 3330 participants (64.9%) performed a quantitative test and the positivity rates for the immunoglobulin tested were 5.6% for IgM (186/3330 positives) and 14.9% for IgG (481/3222 positives), with 122 being positive to both IgM and IgG. Among the remaining subjects who performed the rapid qualitative test, the seroprevalence was 0.7% for IgM (13/1797 positives) and 1.4% for IgG (25/1797 positives) with three being positive to both IgM and IgG. The subjects who performed the quantitative test showed the highest seroprevalence. The percentage of seropositivity is higher among self-referred subjects compared to employer-referred subjects.

Table 3 and Table S2 show the seroprevalence based on occupational category. The highest seroprevalence was observed in the group of healthcare workers (22.9% of the entire category), followed by “information and communication services; financial and insurance activities; professional scientific and technical activities: rental, travel agencies, business support services” (15.7%) and workers in the sports sector (5.2%). A high seroprevalence was also found in the group of “other service activities” that includes spa activities and services for physical well-being and repair of household appliances. No seropositivity emerged in the “constructions”, “activities of the accommodation and restaurant services”, “agriculture, forestry and fishing”, “water supply; sewer networks, waste management and remediation activities”, and “education” sectors.

## 4. Discussion

This study evaluated the seroprevalence of anti-SARS-CoV-2 antibodies in a population of participants in Northern Italy from September 2020 to March 2021 in order to estimate the size and extent of the infection after the first pandemic wave, taking into account sociodemographic characteristics, namely sex, age, province of origin, and professional activity. Overall, the study population demonstrated an overall percentage of infected people of approximately 11%. These data highlighted a substantial increase compared to the first Italian national seroprevalence SARS-CoV-2 antibody survey carried out for the period from 25 May–15 July 2020, which showed a seroprevalence of 2.5% in Italy and 2.8% in the Emilia-Romagna region [19]. Our estimate is also higher compared to the global estimation of 4.5% provided in a previous systematic review and meta-analysis that synthesized seroprevalence data from 74 countries, with a population of 9.3 million in data reported from 1 January to 31 December 2020 [16]. However, some variation in seroprevalence was also noted in our study, in particular, depending on the type of test used, from 2% for qualitative tests to 16% for quantitative serological tests. This difference could be related to the preferential use of quantitative tests in case of suspected infection, due to their higher sensibility compared to qualitative tests [25]. In particular, the percentage of seroprevalence was found to be 14.9% for only the IgG antibodies from quantitative tests. Other studies carried out in Italy have reported a value of seroprevalence comparable with our results from quantitative tests, especially in the areas in the North of the country. Valenti and colleagues investigated the trends and risk factors of SARS-CoV-2 infection in blood donors. A total of 8798 healthy blood donors from Milan were selected from July 2020 to February 2021. The estimated seroprevalence was approximately 4% in early July 2020 and remained stable over the summer. Conversely, it began to increase in November 2020 during the second wave of SARS-CoV-2 infection, before the start of the vaccination campaign, reaching a seroprevalence of around 15% by the end of February 2021 [26].

A comparison of the present findings with the previous study was carried out in the same area in the period from June–September 2020, after the first wave showed that the percentage of seropositive individuals who performed a quantitative serological test was approximately 5.8% compared to the 16.4% of the most recent investigation, although it was based on a different number of participants [17]. However, there are several international studies that have shown a different seroprevalence than that which emerged from the Italian seroprevalence survey [19]. A Scottish study found an estimated seroprevalence of 9.6% in December 2020 [27]. This finding is supported by the seroprevalence estimates of other countries at similar stages of the pandemic [28]. A Swedish survey carried out in blood donors and pregnant women showed approximately 15% seroprevalence for the period of December 2020 [29]. Similar data were reported in a Portuguese cross-sectional study, with seroprevalence of 13.5% between February and March 2021 among the 8463 participants aged 1 to 79 [30].

Interestingly, we found a higher seroprevalence in women than men (12.5% vs. 10.8%). This is consistent with other studies that analyzed the sex difference in antibodies response [31].

Our results also showed a different distribution by age group, with higher seroprevalence in older individuals, particularly those aged 60–69 years and >70 years (18.6% and 18.0%, respectively). In France, during the first wave, the risk of becoming a case was higher for contacts aged 60–74 years (adjusted odds ratio-AOR: 2.0, 95% CI: 1.2–3.3), and older than 75 years (AOR: 2.1, 95% CI: 1.1–3.9), compared with the reference group of 15–29 years [32]. Further relevant data concerned the high seroprevalence of individuals aged <20 years (17.1%). This finding could be justified by both the increase in infections in the younger age group and the increased availability of test and identification of cases among the younger population, more frequently pauci-symptomatic or asymptomatic. Moreover, the reduction of restrictive measures may have contributed to the increased spread of the virus, especially among younger individuals [14].

Several previous studies have assessed the antibody response in healthcare workers and have identified that category as the one most exposed to SARS-CoV-2 infection [33,34,35,36,37]. This result is not surprising due to a high seroprevalence of anti-SARS-CoV-2 antibodies already identified in healthcare workers, especially those in Northern Italy heavily affected by the pandemic, particularly in the first pandemic wave [38,39]. Our study results show a seroprevalence of 22.9% in the health sector and confirm it as the category with the highest seroprevalence. The corresponding value concerning our study of seroprevalence of anti-SARS-CoV-2 antibodies in a population residing in the province of Modena in the period from June–September 2020 was 8.8% [17]. In the literature, there are meta-analyses that have served to synthesize data from different countries in order to better understand the distribution of SARS-CoV-2 infection among healthcare workers [40,41]. Hossain et al. selected a total of 53 articles published from 1 January 2020 to 15 January 2021, including 173,353 healthcare workers from the United States, 10 European countries, and 3 from East Asia. The overall measure of the seroprevalence rate of IgG antibodies was 8.6% in those regions (95% confidence interval-CI: 7.2–9.9%). The aggregate seroprevalence of IgG antibodies was higher in the studies made in the USA (12.4%, 95% CI: 7.8–17%) compared to those in Europe (7.7%, 95% CI: 6.3–9.2%) and East Asia (4.8%, 95% CI: 2.9–6.7%) [40], denoting a wide variation in the seroprevalence data across different countries, possibly related to demographic (e.g., age, sex, ethnicity) and socioeconomic characteristics of the different healthcare workers [40]. This highlights the high risk of infection for this category and the need to increase protection for workers in close contact with patients [42]. Based on these results, it seemed reasonable to use the available resources to carry out screening campaigns of healthcare workers at higher risk of infection. In settings with limited resources, it was preferred to focus testing on symptomatic healthcare workers to maximize efficacy considering their continued exposure. However, a significant number of workers were infected but showed no symptoms. Therefore, in medium and high resource settings, mass screening for all healthcare workers exposed to confirmed cases of COVID-19 was the best approach to limit the spread of the virus [18,43].

Other occupational activities appear to be more at risk of SARS-CoV-2 infection. Workers in direct contact with customers, such as information and communication services, financial and insurance activities, rental agencies, travel agencies, business support services, but also those in the sports sector experienced a higher seroprevalence of anti-SARS-CoV-2 antibodies. On the other hand, we found that no excess of seropositivity emerged for workers in the manufacturing and construction sectors and for transport and storage workers. Interestingly, no increased risk was found for workers in the restaurant and education sector. The national lockdown periods have guaranteed mobility and distancing measures to make contagion less likely in these sectors. Additionally, among the exit strategies from lockdown, several countries, including Italy, implemented and improved the use of distance learning and physical distancing in school settings [44], as well as several recommendations provided for foodservice reopening and organization of living spaces [45,46]. An important seroprevalence of anti-SARS-CoV-2 antibodies emerged in the occupational groups of workers in the sports sector. This interesting discovery confirmed the value found in our previous studies of workers in the province of Modena carried out in the period from June–September 2020, showing the risk caused by contact sports and the increased risk of viral transmission by air [17,47]. The interruption of amateur and recreational sport has caused significant global implications, in the economic, social, and health aspects of the population’s well-being [48]. In this regard, the Italian Federation of Sports Medicine (FMSI) disseminated recommendations for the resumption of competitions and training. For this reason, in April 2020, indications were provided on the protocol to be followed for the medical sports evaluation of professional and amateur athletes with previous SARS-CoV-2 infection [49].

This study has some limitations. We were not able to collect all the information regarding the characteristics of the SARS-CoV-2 infection, such as the health conditions of the individuals, whether the participants were asymptomatic or presented COVID-19 symptoms, nor about virus type (wild-type virus or variants), duration, and severity. These data would have been useful to evaluate the extent and duration of the immune response against SARS-CoV-2. However, the anti-SARS-CoV-2 humoral immunity seems to last for at least 1 year in most convalescents, protecting them from the original virus, as reported by Liu et al. [50]. Considering that most subjects were from Modena province, the reliability of seroprevalence estimates stratified by province may be low, especially considering the small sample size. We did not know if the infection was ongoing or past at the time of sample collection, as IgG serological assays seem to be a more reliable tool for the retrospective diagnosis of SARS-CoV-2 infection [51]. Despite none of the study subjects underwent mandatory serological investigation, the different reasons for testing, i.e., self-referral and employer referral, limit the generalization of results to the overall population of Modena province, as well as a thorough comparison of seroprevalence rates between the two groups (workers and non-workers) in the same period and over time, especially considering working activities. Finally, the limited sample size did not allow us to perform stratified analysis for some working categories.

This study also has some strengths. It provides seroprevalence data stratified by sex and it complements a seroprevalence study performed on workers in the province of Modena in an earlier period, thus providing further information on the spread of the virus in this area over the first two pandemic waves. Moreover, the information on the employment status of the study participants made it possible to provide ideas for the implementation of further preventive measures in the workplace and for the more accurate identification of infection transmission.

## 5. Conclusions

This study provided epidemiological data of anti-SARS-CoV-2 seroprevalence in a population of participants in Emilia-Romagna, a region heavily affected by the pandemic since its onset. The results showed that the occupational categories with the highest risk of exposure to SARS-CoV-2 were healthcare workers, workers in the sports sector, and other activities, such as information and communication services, financial and insurance activities, rental agencies, travel agencies, and business support services.

## Figures and Tables

**Table 1:** Characteristics of 5128 study participants recruited in the period of September 2020–March 2021, at the Test laboratory in Modena, Italy. Data are shown in number (*N*) and percentage (%) where not differently reported.

Characteristics	Total	Men	Women
	*N* (%)	*N* (%)	*N* (%)
Overall	5128 (100)	3124 (60.9)	2004 (39.1)
Age (years) ^a^	43.5 ± 14.8	43.0 ± 14.6	44.3 ± 15.1
<65 years	4703 (91.7)	2893 (92.6)	1810 (88.7)
≥65 years	425 (8.3)	231 (7.4)	194 (11.3)
Province of residence			
Modena	4038 (78.7)	2392 (76.6)	1646 (82.1)
Reggio Emilia	114 (2.2)	61 (2.0)	53 (2.6)
Parma	9 (0.2)	2 (0.1)	7 (0.3)
Bologna	20 (0.4)	10 (0.3)	10 (0.5)
other/missing data	947 (18.5)	659 (21.1)	288 (14.4)
Referral category			
workers	3889 (76.8)	2559 (82.0)	1330 (66.4)
private	1239 (24.2)	565 (18.0)	674 (33.6)
Occupational sector (workers only)			
agriculture, forestry and fishing (A)	5 (0.1)	3 (0.1)	2 (0.1)
manufacturing activities (C)	1182 (30.4)	787 (30.8)	395 (29.7)
water supply; sewer networks, waste management and remediation activities (E)	7 (0.2)	5 (0.2)	2 (0.1)
constructions (F)	38 (1.0)	25 (1.0)	13 (1.0)
wholesale and retail trade; repair of motors vehicles and motorcycles (G)	270 (6.9)	173 (6.8)	97 (7.3)
transport and storage (H)	42 (1.1)	27 (1.1)	15 (1.1)
activities of the accommodation and restaurant services (I)	1 (0.03)	1 (0.04)	-
information and communication services; financial and insurance activities; professional scientific and technical activities; rental, travel agencies, business support services (J, K, M, N)	1256 (32.3)	862 (33.7)	394 (29.6)
education (P)	2 (0.05)	2 (0.1)	-
health sector (Q)	573 (14.7)	265 (10.4)	308 (23.1)
workers in the sports sector (R)	441 (11.3)	382 (14.9)	59 (4.4)
other service activities (S)	72 (1.8)	27 (1.1)	45 (3.4)

Notes: ^a^ mean (standard deviation).

**Table 2:** Anti-SARS-CoV-2 antibody positive (Ab+) tests in the period of September 2020–March 2021 at the Test laboratory in Modena, Italy. Overall, 5128 participants. Data are shown in number (*N*) and percentage (%).

	Total		Men		Women	
	Total Test	Ab+ Test	Total Test	Ab+ Test	Total Test	Ab+ Test
	*N* (%)	*N* (%)	*N* (%)	*N* (%)	*N* (%)	*N* (%)
Overall	5128 (100)	580 (11.3)	3124 (60.9)	333 (10.7)	2004 (39.1)	247 (12.3)
Age						
<20 years	123 (2.4)	21 (17.1)	73 (2.3)	10 (13.7)	50 (2.5)	11 (22.0)
20–29 years	929 (18.1)	71 (7.6)	590 (18.9)	37 (6.3)	339 (16.9)	34 (10.0)
30–39 years	1048 (20.4)	76 (7.3)	657 (21.0)	44 (6.7)	391 (19.5)	32 (8.2)
40–49 years	1276 (24.9)	142 (11.1)	767 (24.6)	86 (11.2)	509 (25.4)	56 (11.0)
50–59 years	1054 (20.6)	142 (13.5)	637 (20.4)	89 (14.0)	417 (20.8)	53 (12.7)
60–69 years	431 (8.4)	80 (18.6)	256 (8.2)	43 (16.8)	175 (8.7)	37 (21.1)
≥70 years	267 (5.2)	48 (18.0)	144 (4.6)	24 (16.7)	123 (6.1)	24 (19.5)
Test type						
Quantitative	3330 (64.9)	545 (16.4)	2050 (65.5)	309 (15.1)	1280 (63.8)	236 (18.4)
Qualitative	1798 (35.1)	35 (2.0)	1074 (34.5)	24 (2.2)	724 (36.2)	11 (1.5)
Antibody/Ig tested						
IgG	5019 (97.9)	506 (10.1)	3063	292 (9.5)	1956	214 (10.9)
IgM	5128 (100)	199 (3.9)	3124	120 (3.8)	2004	79 (3.9)
Referral category						
workers	3889 (76.8)	409 (10.5)	2559 (82.0)	254 (9.9)	1330 (66.4)	155 (11.7)
private	1239 (24.2)	171 (13.8)	565 (18.0)	79 (14.0)	674 (33.6)	92 (13.7)

**Table 3:** Anti-SARS-CoV-2 antibody (Ab) status and percentage of antibody positivity by occupational category using ATECO classification in workers in the period of September 2020–March 2021, at the Test laboratory in Modena, Italy.

	Total (*N* = 3889)		Men (*N* = 2559)		Women (*N* = 1330)	
	Ab+/Test Tot	Ab+	Ab+/Test Tot	Ab+	Ab+/Test Tot	Ab+
Occupational Sector	N/N	%	N/N	%	N/N	%
agriculture, forestry and fishing (A)	0/5	0.0	0/3	0.0	0/2	0.0
manufacturing activities (C)	39/1182	3.3	31/787	3.9	8/395	2.0
water supply; sewer networks, waste management and remediation activities (E)	0/7	0.0	0/5	0.0	0/2	0.0
constructions (F)	0/38	0.0	0/25	0.0	0/13	0.0
wholesale and retail trade; repair of motors vehicles and motorcycles (G)	13/270	4.8	7/173	4.0	6/97	6.2
transport and storage (H)	1/42	2.4	1/27	3.7	0/15	0.0
activities of the accommodation and restaurant services (I)	0/1	0.0	0/1	0.0	-	-
information and communication services; financial and insurance activities; professional scientific and technical activities; rental, travel agencies, business support services (J, K, M, N)	197/1256	15.7	131/862	15.2	66/394	16.8
education (P)	0/2	0.0	0/2	0.0	-	-
health sector (Q)	131/573	22.9	65/265	24.5	66/308	21.4
workers in the sports sector (R)	23/441	5.2	18/382	4.7	5/59	8.5
other service activities (S)	5/72	6.9	1/27	3.7	4/45	8.9

## Data Availability

The data presented in this study are available on reasonable request from the corresponding author. The data are not publicly available due to privacy and legal restrictions.

## Supplementary Materials

The following supporting information can be downloaded at: https://www.mdpi.com/article/10.3390/ijerph19137882/s1, Table S1: Anti-SARS-CoV-2 antibody negative (Ab−) and positive (Ab+) tests in the period of September 2020–March 2021 at the test laboratory in Modena, Italy. Overall, 5128 participants. Data are shown in number (N) and percentage (%); Table S2: Anti-SARS-CoV-2 antibody (Ab) status and percentage of antibody negativity (Ab−) and positivity (Ab+) by occupational category using ATECO classification in workers in the period of September 2020–March 2021, at the test laboratory in Modena, Italy.
