# Supplementary material for: Seroprevalence Survey of Anti-SARS-CoV-2 Antibodies in a Population of Emilia-Romagna Region, Northern Italy
Source: Int J Environ Res Public Health. 2022 Jun 27;19(13):7882. doi: 10.3390/ijerph19137882 (PMC9266015; doi:10.3390/ijerph19137882)
Supplement: Supplementary file 1 [file ijerph-19-07882-s001.zip › ijerph-1737007-supplementary.pdf]

Table S1. Anti-SARS-CoV-2 antibody negative (Ab-) and positive (Ab+) tests in the period of September 2020-March 2021 at the Test Laboratory in Modena, Italy. Overall 5128 participants. Data are number (N) and percentage (%).

	**Total**		**Men**		**Women**	
	**Ab- test**	**Ab+ test**	**Ab- test**	**Ab+ test**	**Ab- test**	**Ab+ test**
	**N (%)**	**N (%)**	**N (%)**	**N (%)**	**N (%)**	**N (%)**
Overall	4548 (88.7)	580 (11.3)	2791 (89.3)	333 (10.7)	1757 (87.7)	247 (12.3)
Age						
< 20 years	102 (82.9)	21 (17.1)	63 (86.3)	10 (13.7)	39 (78.0)	11 (22.0)
20-29 years	858 (92.4)	71 (7.6)	553 (93.7)	37 (6.3)	305 (90.0)	34 (10.0)
30-39 years	972 (92.7)	76 (7.3)	613 (93.3)	44 (6.7)	359 (91.8)	32 (8.2)
40-49 years	1134 (88.9)	142 (11.1)	681 (88.8)	86 (11.2)	453 (89.0)	56 (11.0)
50-59 years	912 (86.5)	142 (13.5)	548 (86.0)	89 (14.0)	364 (87.3)	53 (12.7)
60-69 years	351 (81.4)	80 (18.6)	213 (83.2)	43 (16.8)	138 (78.9)	37 (21.1)
≥70 years	219 (82.0)	48 (18.0)	120 (83.3)	24 (16.7)	99 (80.5)	24 (19.5)
Test type						
Quantitative	2785 (83.6)	545 (16.4)	1741 (84.9)	309 (15.1)	1044 (81.6)	236 (18.4)
Qualitative	1763 (98.0)	35 (2.0)	1050 (97.8)	24 (2.2)	713 (98.5)	11 (1.5)
Antibody/Ig tested						
IgG	4513 (89.9)	506 (10.1)	2771 (90.5)	292 (9.5)	1742 (89.1)	214 (10.9)
IgM	4929 (96.1)	199 (3.9)	3004 (96.2)	120 (3.8)	1925 (96.1)	79 (3.9)
Referral category						
workers	3480 (89.5)	409 (10.5)	2305 (90.1)	254 (9.9)	1175 (88.3)	155 (11.7)
private	1068 (86.2)	171 (13.8)	486 (86.0)	79 (14.0)	582 (86.3)	92 (13.7)

Table S2. Anti-SARS-CoV-2 antibody (Ab) status and percentage of antibody negativity (Ab-) and positivity (Ab+) by occupational category using ATECO classification in workers in the period of September 2020-March 2021, at the Test Laboratory in Modena, Italy.

	**Total (N=3889)**		**Men (N=2559)**		**Women (N=1330)**	
	**Ab- test**	**Ab+ test**	**Ab- test**	**Ab+ test**	**Ab- test**	**Ab+ test**
**Occupational sector**	**N (%)**	**N (%)**	**N (%)**	**N (%)**	**N (%)**	**N (%)**
agriculture, forestry and fishing (A)	5 (100)	0 (0.0)	3 (100)	0 (0.0)	2 (100)	0 (0.0)
manufacturing activities (C)	1143 (96.7)	39 (3.3)	756 (96.1)	31 (3.9)	387 (98.0)	8 (2.0)
water supply; sewer networks, waste management and remediation activities (E)	7 (100)	0 (0.0)	5 (100)	0 (0.0)	2 (100)	0 (0.0)
constructions (F)	38 (100)	0 (0.0)	25 (100)	0 (0.0)	13 (100)	0 (0.0)
wholesale and retail trade; repair of motors vehicles and motorcycles (G)	257 (95.2)	13 (4.8)	166 (96.0)	7 (4.0)	91 (93.8)	6 (6.2)
transport and storage (H)	41 (97.6)	1 (2.4)	26 (96.3)	1 (3.7)	15 (100)	0 (0.0)
activities of the accommodation and restaurant services (I)	1 (100)	0 (0.0)	1 (100)	0 (0.0)	-	-
information and communication services; financial and insurance activities; professional scientific and technical activities; rental, travel agencies, business support services (J, K, M, N)	1059 (84.3)	197 (15.7)	731 (84.8)	131 (15.2)	328 (83.2)	66 (16.8)
education (P)	2 (100)	0 (0.0)	2 (100)	0 (0.0)	-	-
health sector (Q)	442 (77.1)	131 (22.9)	200 (75.5)	65 (24.5)	242 (78.6)	66 (21.4)
workers in the sports sector (R)	418 (94.8)	23 (5.2)	364 (95.3)	18 (4.7)	54 (91.5)	5 (8.5)
other service activities (S)	67 (93.1)	5 (6.9)	26 (96.3)	1 (3.7)	41 (91.1)	4 (8.9)
